# Interleukin-10 attenuates impairment of the blood-brain barrier in a severe acute pancreatitis rat model

**DOI:** 10.1186/s12950-018-0180-0

**Published:** 2018-02-27

**Authors:** Ronggui Lin, Fei Chen, Shi Wen, Tianhong Teng, Yu Pan, Heguang Huang

**Affiliations:** Department of General surgery, Fujian Medical University Union Hospital, Fuzhou, Fujian People’s Republic of China

**Keywords:** IL-10, Blood-brain barrier, Endothelial cell, Tight junction, Apoptosis

## Abstract

**Background:**

Impairment of the blood-brain barrier (BBB) in severe acute pancreatitis (SAP) could result in life-threatening pancreatic encephalopathy. Interleukin-10 (IL-10) is a classical cytokine that is well-known for its strong immunoregulatory and anti-inflammatory abilities. However, whether and how IL-10 protects the BBB in SAP are still unclear.

**Methods:**

This study includes in vivo experiments using a SAP rat model and in vitro experiments using an in vitro BBB model consisting of a monolayer of brain microvascular endothelial cells (BMECs). The study groups are divided into the control, SAP (in vivo)/TNF-α (in vitro), IL-10 treatment, IL-10 + signal transducer and activator of transcription 3 (STAT3) inhibitor S3I-201 treatment groups. Pancreatic pathological scores, serum amylase, serum TNF-α levels and BBB permeability by Evan’s blue assay in SAP rat models were evaluated. BMEC apoptosis in SAP rats or induced by TNF-αin vitro was detected by terminal-deoxynucleotidyl transferase-mediated nick end labeling (TUNEL) and flow cytometry, separately. Expression levels of claudin-5 and proteins involved in the STAT3 signaling pathway were measured by Western blotting. Location and changes of junctional structure of claudin-5 on BMECs were assessed by immunohistochemistry and immunofluorescence.

**Results:**

In vivo, IL-10 alleviated the severity of inflammation, attenuated the increased BBB permeability in SAP rat models by reducing BMEC apoptosis via the STAT3 pathway and ameliorated the down-regulation of claudin-5 expression in BMECs; in vitro, IL-10 improved BBB integrity against TNF-α by attenuating BMEC apoptosis via the STAT3 pathway, the impairment of tight junction structure and the down-regulation of claudin-5 expression in BMECs.

**Conclusions:**

IL-10 improves BBB properties in SAP by attenuating the down-regulation of claudin-5 expression and the impairment of tight junctions and by STAT3 pathway-mediated anti-apoptotic effects on BMECs.

## Background

Severe acute pancreatitis (SAP) is a critical acute abdominal disease characterized by an acute onset and a rapid progression, with a mortality rate up to 10–25% [1]. Pancreatic encephalopathy is a serious complication in SAP and is associated with a mortality rate of 67% [2]. Though the pathogenesis is unclear, a close relationship between pancreatic encephalopathy and impairment of the blood-brain barrier (BBB) is commonly accepted. Published reports have demonstrated increased BBB permeability in SAP [3]. Consequently, improving the barrier properties of the BBB against inflammation is of great significance in preventing and treating pancreatic encephalopathy in the clinical setting.

The BBB is mainly composed of brain microvascular endothelial cells (BMECs), astrocytes and pericytes [4]. BMECs and peri-cellular tight junctions are basic and functional characteristics of the BBB. Severe inflammation has been shown to induce BMEC apoptosis, and impairment of peri-cellular tight junctions result in BBB dysfunction [5]. Hence, attenuating BMEC apoptosis and the deterioration of tight junctions is beneficial for stabilizing the BBB. As a classical anti-inflammatory cytokine, interleukin-10 (IL-10) suppresses local and systematic inflammation and exhibits immunoregulatory and anti-apoptotic abilities [6]. A previous study showed that IL-10 reduced brain endothelial cell apoptosis in bacteremia [7]. However, whether IL-10 alleviates BMEC apoptosis and the impairment of peri-cellular tight junctions in SAP remains unclear.

The signal transducer and activator of transcription 3 (STAT3) signaling pathway is widely known to be involved in inflammatory and anti-inflammatory responses, tumor progression and cell autophagy [8]. The STAT3 pathway mediates the inflammatory response when activated by IL-6, but it mediates the anti-inflammatory response when activated by IL-10 [9]. STAT3 is phosphorylated and translocated to the nucleus to modulate downstream target protein expression following activation of the STAT3 pathway. Phosphorylated STAT3 (p-STAT3) has been reported to reduce endothelial cell apoptosis in mouse atherosclerosis models [10] but increase heme-induced endothelial cell apoptosis by up-regulating the target gene matrix metalloproteinase 3 in another study [11]. Based on the hypothesis that IL-10 reduces BMEC apoptosis in SAP, whether the STAT3 pathway is involved in anti-apoptotic effects remains unclear.

In the present study, we established a SAP rat model as well as an in vitro BBB model by exposure to TNF-α; then, we evaluated the effects of IL-10 and STAT3 inhibitor (S3I-201) administration. We found that IL-10 attenuated BMEC apoptosis, the impairment of peri-cellular tight junctions and the down-regulation of the tight junction protein claudin-5 under inflammatory conditions. Meanwhile, we found that IL-10 induced anti-apoptotic effects via the STAT3 pathway by decreasing Bcl-2-associated X (Bax) expression while increasing B-cell lymphoma 2 (Bcl-2) expression in BMECs.

## Methods

### Ethics

All animal experiments were approved by the Experimental Animal Committee of Fujian Medical University and performed strictly according to the guidelines of the National Institutes of Health (NIH Publications No. 80–23, revised in 1996). All animals received humane care and were fed under standard conditions.

### Animals

Clean adult male Sprague-Dawley rats weighing 200–250 g were purchased from Shanghai SLAC Laboratory Animal Co. Ltd. (China). The rats were raised in rooms with a 12-h light/dark cycle at 25 °C for at least 1 week. A total of 32 rats were randomly divided into the control group, SAP group, SAP+IL-10 treatment group and SAP+IL-10 + S3I-201 treatment group (*n* = 8 per each group). All the rats were sacrificed 12 h later for sample collection.

### Establishment of a SAP rat model

SAP rat models were established via retrograde injection of 5% sodium taurocholate as previously described [12, 13]. The rats were fasted 12 h before the procedure but were allowed to drink water. A 10% chloral hydrate solution (3 ml/kg body weight, Bio Basic, Canada) was injected into the abdominal cavity as anesthesia. Laparostomy was performed through a 1–1.5-cm incision in the midline of the upper abdomen. Then, 5% sodium taurocholate (Inalco Spa, Milano, Italy) was injected into the biliopancreatic duct at 1 ml/kg body weight using a 0.45-mm diameter polyethylene catheter attached to a microinfusion pump at a speed of 0.04 ml/min. The catheter was removed 10 min later, and the abdomen was closed with sutures. In the control group, the duodenum and pancreas were maneuvered without the injection into the biliopancreatic duct. All the procedures were performed under aseptic conditions. The rats received 4 mL/100 g body weight of normal saline every 6 h by subcutaneous injection in the back.

### Pancreatitic pathological scores

After fixing in 10% formalin solution for more than 24 h, the collected body of pancreases were embedded in paraffin and sectioned at 4-μm thickness followed by staining with hematoxylin and eosin (H&E). Pathological changes in the pancreas were evaluated with the pathological score system [14] under a light microscope (Carl Zeiss, Germany).

### Serum amylase analysis and enzyme-linked immunosorbent assay (ELISA)

Collected blood samples were centrifuged at 3000 x g at 4 °C for 10 min, and the acquired serum was stored at − 80 °C before analysis. Serum amylase was detected using Olympus AV2700 automated clinical biochemistry analysis equipment (Olympus, Tokyo, Japan). Serum levels of TNF-α were detected using a rat ELISA kit (R&D Systems, USA) according to the manufacturer’s instructions.

### Evan’s blue assay

The Evan’s blue assay was performed to evaluate BBB permeability in SAP rats as described in a previous study [15]. A 2% Evan’s blue solution (5 mL/kg body weight, Sigma, USA) was injected via the femoral vein 1 h before sacrificing the rats. The rats were perfused with cold phosphate buffered saline (PBS) to clear the Evan’s blue solution from the vessels. Then, 300 mg of brain tissue was incubated in 1 mL of dicarboamide and homogenized by an ultrasonic cell disruptor. Then, another 3 mL of dicarboamide was added and the samples were incubated at 37 °C for 48 h and then centrifuged at 300×g for 5 min. The acquired supernatants were detected using a SpectraMax M5e Multi-Mode Microplate Reader (Molecular Devices, USA) (absorbance at 620 nm).

### Western blot assay

Western blot assays were performed to evaluate Claudin-5, p-STAT3, STAT3, Bax and Bcl-2 expression levels in the brain of SAP rats and in in vitro BBB models. Brain tissue was homogenized on ice, and BMECs on the Transwell insert were scraped and then added to ice-cold RIPA lysis buffer (Beyotime, China) containing 1 mM PMSF (Beyotime). The supernatants were preserved after centrifugation at 14,000×g at 4 °C for 10 min, and their protein concentrations were measured with a bicinchoninic acid (BCA) assay kit (Beyotime). Approximately 50 μg of total protein was separated by SDS PAGE using a proper concentration of SDS and transferred to nitrocellulose (NC) membranes. The membranes were blocked with 5% skim milk for 1 h and then incubated with the following primary antibodies at 4 °C overnight: mouse anti-claudin-5 (1:500, Catalog No. 35–2500, Invitrogen, USA), rabbit anti-p-STAT3 (1:1000, Catalog No.9145, Cell Signaling Technology, USA), rabbit anti-STAT3 (1:1000, Catalog No.12640, Cell Signaling Technology), rabbit anti-Bcl-2 (1:1000, Catalog No.ab59348, Abcam, USA), rabbit anti-Bax (1:1000, Catalog No.2772, Cell Signaling Technology), and mouse anti-β-actin (1:1000, Catalog No.HC201–02, Transgen, China). Afterwards, the membranes were incubated with corresponding secondary antibodies: goat anti-mouse IgG-HRP and goat anti-rabbit IgG-HRP antibodies (1:5000, Cell Signaling Technology) at room temperature for 2 h. The immunoglobulins were then detected with a ChemiDocTM MP imaging system (Bio-Rad, USA) using the enhanced chemiluminescence (ECL) technique. The western blot (WB) results were analyzed using ImageJ software (version 1.48u).

### Immunohistochemistry

Immunohistochemistry was carried out to assess the expression of claudin-5 in the brain of SAP rats. Brain samples were fixed in 4% paraformaldehyde for over 24 h, embedded and sectioned at 5-μm thickness. After dewaxing, rehydration and antigen retrieval, the sections were preincubated with 3% hydrogen peroxide to inactivate endogenous peroxidase and blocked with 5% bovine serum albumin (BSA, Sigma) for 1 h. The sections were then incubated with a mouse anti-claudin-5 (1:50, Invitrogen) primary antibody at 4 °C overnight followed by a secondary antibody (1:100; Abcam) at 37 °C for 30 min. Peroxidase activity was revealed by 3-diaminobenzidine (DAB). After staining with hematoxylin, the sections were dehydrated in an alcohol gradient, cleared with xylene and coverslipped.

### Terminal-deoxynucleotidyl transferase-mediated nick end labeling (TUNEL) assay

TUNEL assay was performed to evaluate BMEC apoptosis in the brain of SAP rats using a TUNEL staining kit (POD, Roche, USA). After dewaxing, rehydration and inactivation of endogenous peroxidase, the sections were incubated with TUNEL reaction mixture at 37 °C for 60 min. The nuclei were stained with DAPI (1:1000, Sigma). TUNEL-positive and DAPI-positive cells were counted under a fluorescence microscope (Carl Zeiss).

### Cell culture of BMECs

Primary BMECs were extracted with a previously described method [16]. A clean 1-month-old Sprague-Dawley rat was anesthetized and decapitated under aseptic conditions. The brain was placed into ice-cold PBS, and the brain cortices were preserved after the meninges were removed. Minced gray matter was digested with 15 mg/ml DNase I (Sigma) and 1 mg/ml collagenase type 2 (Worthington, USA) in Dulbecco’s modified Eagle’s medium (DMEM, HyClone, USA) at 37 °C for 1.5 h. The acquired pellet was then centrifuged (1000×g, 4 °C) in 20% BSA for 20 min. The obtained capillaries were then seeded onto six-well plates precoated with 0.1 mg/ml collagen IV/fibronectin (Sigma) and incubated at 37 °C with 5% CO2/95% air. The BMEC culture medium contained 20% fetal bovine serum (Gibco, USA), 5 μg/ml insulin (Sigma), 100 μg/ml heparin (Sigma), 1 ng/ml basic fibroblast growth factor (PeproTech, USA) and 1% penicillin/streptomycin (HyClone) in DMEM. Puromycin (4 mg/L, Sigma) [17] was administered to eliminate the contaminating cells in cultured BMECs and was removed 2 days later. BMECs at 80–90% confluence were passaged with 0.125% EDTA-trypsin (Gibco). First-passage BMECs were used for the subsequent experiments.

### Immunofluorescence staining

Immunofluorescence staining was carried out to identify BMECs and to observe the changes in peri-cellular tight junctions and claudin-5 expression. After the BMECs were fixed in 4% paraformaldehyde and then permeabilized with 0.5% Triton X-100 (Sigma) for 15 min, the BMECs were blocked with 5% BSA for 30 min followed by incubation with primary antibodies: rabbit anti-von Willebrand factor (vWF) antibody (1:100, Catalog No. ab6994, Abcam) and mouse anti-claudin-5 antibody (1:50, Invitrogen) at 4 °C overnight. The appropriate secondary antibodies, Alexa Fluor 488-conjugated goat-anti-rabbit or goat-anti-mouse (1:1000, Life Technologies), were then incubated with BMECs at room temperature for 60 min. Nuclei were stained with DAPI (1:1000, Sigma) for 10 min. Cells were observed under a ZEISS LSM 780 confocal microscope (Carl Zeiss, Germany).

### Construction of an in vitro BBB model

First-passage BMECs were incubated at a density of 1.5 × 10^6^ cells/ml on Transwell inserts (1.12 cm^2^, 0.4-μm pore size, Corning 3460, USA) precoated with 0.1 mg/ml collagen IV/fibronectin (Sigma). The culture medium was changed every 2–3 days. The monolayer in vitro BBB model was established 7 days later for further research. The constructed BBB models were divided into the control group, TNF-α group, TNF-α + IL-10 treatment group and TNF-α + IL-10 + S3I-201 treatment group (*n* = 3 per group).

### Transendothelial electrical resistance (TEER) assay

The TEER (Ω × cm2) of the constructed in vitro BBB models was measured to evaluate integrity using a Millicell-ERS electrical resistance system (MER500002, Millipore, USA). Coated cell-free inserts were used as blank groups.

### Flow cytometry analysis

Flow cytometry was used to detect TNF-α-induced BMEC apoptosis. According to the manufacturer’s instructions, BMECs were incubated in 100 μl of incubation buffer with 5 μl of Annexin-V-FITC (Dojindo, Japan) and 5 μl of propidium iodide (PI) protected from light at room temperature for 15 min. Then, the 400 μl of additional incubation buffer was added to the samples and analyzed by flow cytometry analysis (FACS, Beckton Dickinson, USA).

### Drug treatment

IL-10 (PeproTech) was administered to SAP rats at 10 μg/kg body weight through the femoral vein at 0 h and to the constructed BBB models at 20 ng/ml 24 h before TNF-α treatment. S3I-201(Calbiochem, USA) was administered to SAP rats at 5 mg/kg body weight 2 h before the experiment via an intraperitoneal injection and to the constructed BBB models at 100 μM 24 h before IL-10 treatment. TNF-α (20 ng/ml, PeproTech) was administered to the constructed in vitro BBB models for 12 h.

### Statistical analyses

Data were analyzed with the statistical software SPSS 19.0 and Graph Pad Prism 6.0 and presented as the mean ± SE (standard error). All measurements were repeated at least three independent times. t-tests were used to evaluate the differences between groups. A probability (p) value < 0.05 was considered statistically significant.

## Results

### In vivo experiments

#### IL-10 alleviated the severity of inflammation and the increased BBB permeability in SAP rats

**Fig. 1:**
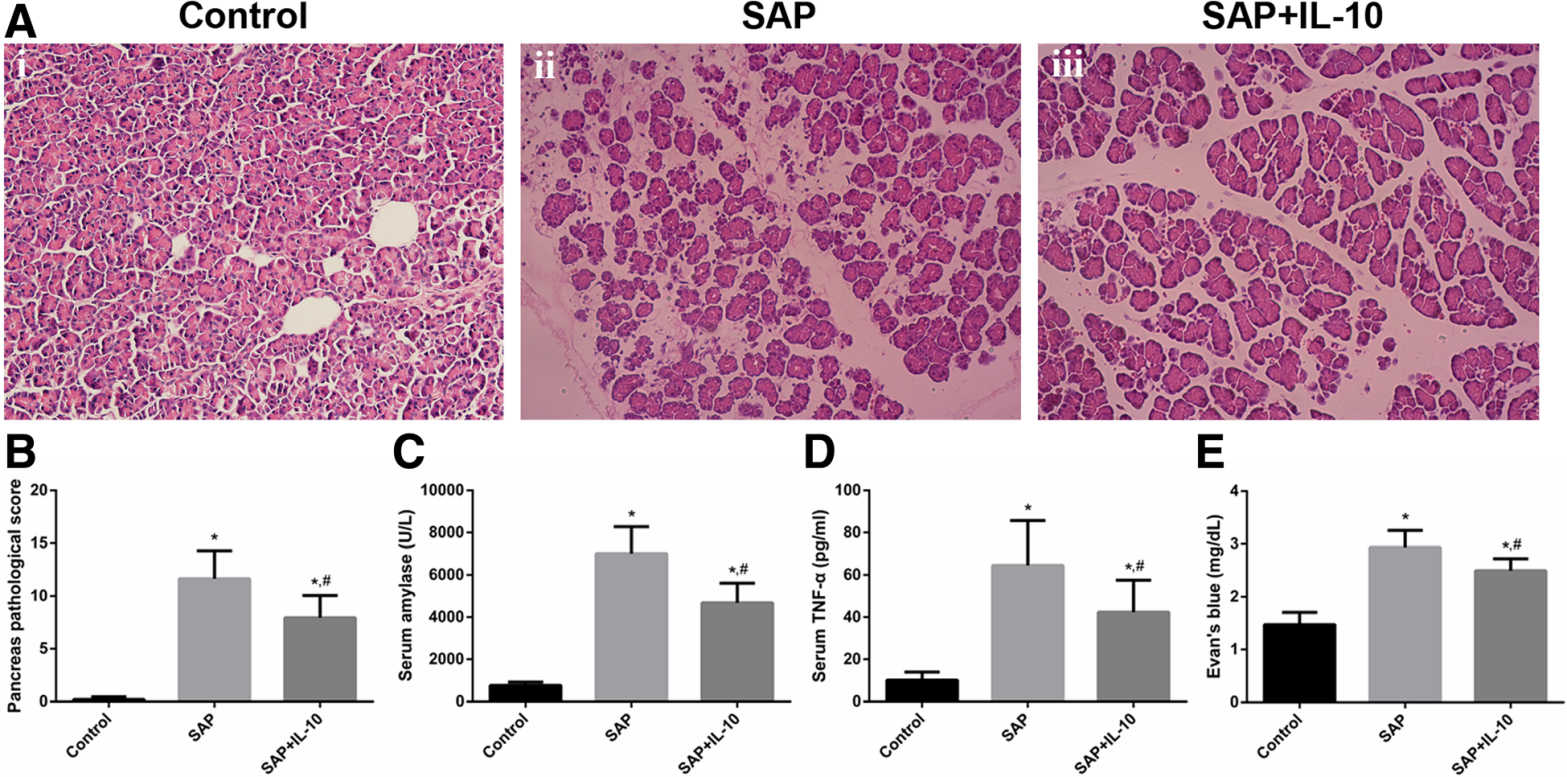
IL-10 alleviated the severity of inflammation and increased BBB permeability in SAP rats. **a** Representative images of H&E-stained rat pancreases (× 200). No significant pathological changes were observed in the pancreases of the control group (i). The pancreases of the SAP group exhibited obvious pancreatic edema, hemorrhage and necrosis as well as inflammatory cell infiltration (ii). Milder impairment of the pancreas was observed in the IL-10 treatment group than in the SAP group (iii). **b** Pancreatic pathological scores (*n* = 8). **c** Serum amylase levels (*n* = 8). **d** Serum TNF-α levels (*n* = 8). E. Evan’s blue assay (*n* = 8). * *p* < 0.001 (t-test), versus the control group, # *p* < 0.05(t-test), versus the SAP group

All rats successfully underwent SAP model establishment, and saponification spots were observed on the mesentery and omentum when the rats were sacrificed. As indicated in Fig. 1a, hematoxylin-eosin staining of the pancreas did not reveal significant pathological changes in the control group. The pancreases of the SAP group exhibited notable pancreatic edema, hemorrhage and necrosis as well as inflammatory cell infiltration. Milder impairment of the pancreas was observed in the IL-10 treatment group than in the SAP group. Both a significantly higher pancreatic pathological score (Fig. 1b) and a significantly higher serum amylase level (Fig. 1c) were observed in the SAP group than in the control group, thus indicating the successful establishment of a SAP rat model. In line with published reports [18], the serum TNF-α level (Fig. 1d) was elevated in the SAP group compared to the control group. Meanwhile, a remarkably higher value of Evan’s blue (Fig. 1e) was observed in the SAP group than in the control group, indicating increased BBB permeability. Additionally, the pancreatic pathological score, serum amylase level, serum TNF-α level and Evan’s blue value were decreased in IL-10-treated SAP rats compared to untreated SAP rats. Taken together, it was inferred that IL-10 reduced the severity of inflammation and the increased BBB permeability in SAP rats.

#### IL-10 attenuated the down-regulation of claudin-5 in the brain of SAP rats

**Fig. 2:**
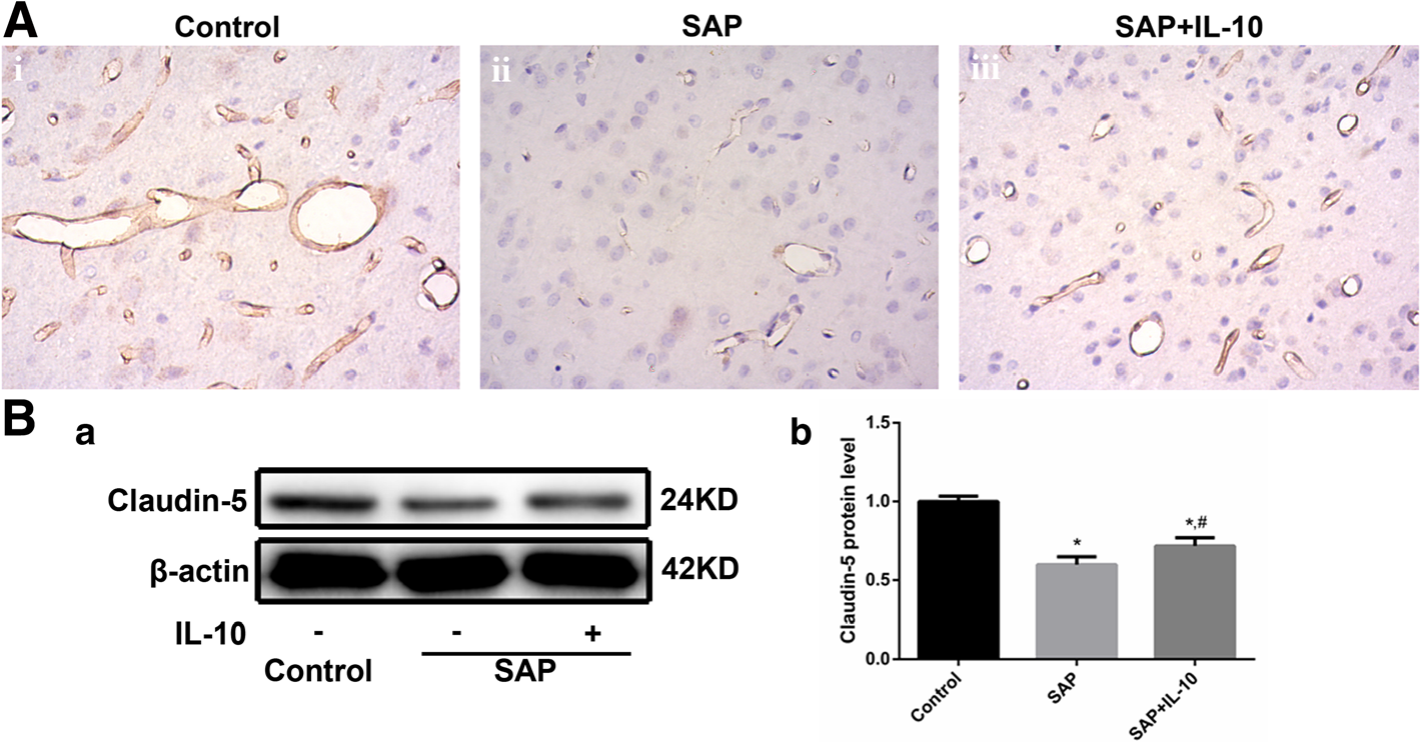
IL-10 attenuated the down-regulation of claudin-5 in the brain of SAP rats. A. Immunohistochemistry of claudin-5 in SAP rat brains (× 400). Control groups exhibit high expression of claudin-5 in BMECs (i). Expression of claudin-5 decreased remarkably in the SAP group compared to the control group (ii). IL-10 attenuated the decreased expression of claudin-5 observed in the SAP group (iii). B. Representative WB images showing claudin-5 expression in SAP rat brains (a). WB analysis of claudin-5 expression (*n* = 8, b). * *p* < 0.01 (t-test), versus the control group, # *p* < 0.05 (t-test), versus the SAP group

To explore the mechanisms underlying the IL-10-mediated alleviation of increased brain permeability in SAP rats, we evaluated claudin-5 expression by WB and immunohistochemistry. Immunohistochemistry (Fig. 2A) showed high expression levels of claudin-5 in control BMECs. Expression of claudin-5 decreased remarkedly in the SAP group compared to the control group. Meanwhile, IL-10 attenuated the decreased expression of claudin-5 in the SAP group. The WB results (Fig. 2B were in accordance with the immunohistochemistry results, showing a noticeable decrease in claudin-5 expression in the SAP group compared with the control group, whereas IL-10 treatment alleviated the decrease in claudin-5 expression. Accordingly, IL-10 attenuated the down-regulation of claudin-5 in the brain of SAP rats, which remarkedly ameliorated brain permeability.

#### IL-10 reduced BMEC apoptosis in SAP rats by the down-regulation of Bax and up-regulation of Bcl-2 expression via the STAT3 pathway

**Fig. 3:**
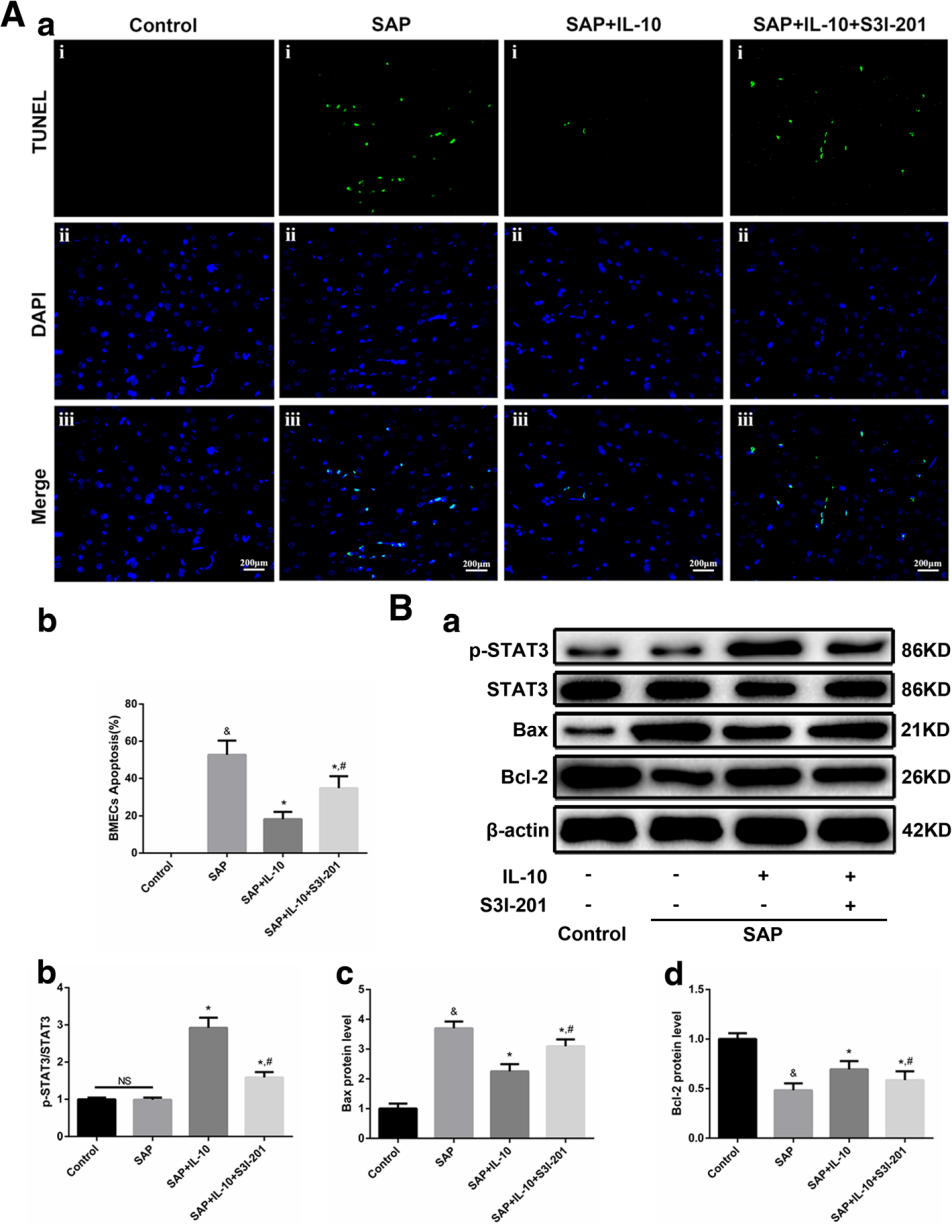
IL-10 reduced BMEC apoptosis in SAP rats via the STAT3 pathway. A. TUNEL staining of BMECs in the rat brain (a); scale bar represents 200 μm. No apoptosis was observed in the control group. The SAP group exhibited numerous apoptotic BMECs and a few apoptotic glial cells. Fewer apoptotic BMECs were observed in either the IL-10 or IL-10 + S3I-201 treatment group than in the SAP group, with the IL-10 group exhibiting the fewest apoptotic BMECs. Statistical graph of BMEC apoptosis as determined by TUNEL staining (*n* = 8, b). B. Representative WB images of p-STAT3, STAT3, Bax and Bcl-2 protein levels in the SAP rat brain (a). WB analysis of p-STAT3/STAT3 expression (*n* = 8, b). WB analysis of Bax expression (*n* = 8, c). WB analysis of Bcl-2 expression (*n* = 8, d). & *p* < 0.001 (t-test), versus the control group, * *p* < 0.05 (t-test), versus the SAP group, # *p* < 0.05 (t-test), versus the SAP+IL-10 treatment group. NS: no statistical difference

To ascertain whether IL-10 attenuates BMEC apoptosis in SAP rats and whether the STAT3 pathway is involved, we administered the STAT3 inhibitor S3I-201. TUNEL staining (Fig. 3A) did not reveal apoptotic cells in the control group, while the SAP group exhibited numerous apoptotic BMECs and a few apoptotic glial cells. Remarkable fewer apoptotic BMECs and glial cells were found in either the IL-10 or IL-10 + S3I-201 treatment group than in the SAP group, with the IL-10 treatment group exhibiting the lowest values. The results of TUNEL staining showed that IL-10 attenuated BMEC apoptosis in SAP rats, thereby exhibiting anti-apoptotic effects, while S3I-201 partly reversed the effects. These results indicated that the STAT3 pathway was involved in the IL-10-induced anti-apoptotic effects.

To explore the molecular mechanisms underlying the IL-10-mediated anti-apoptotic effects on BMECs, we detected the expression of downstream STAT3 pathway proteins, including p-STAT3, STAT3, Bax and Bcl-2. WB results (Fig. 3B) showed that p-STAT3 expression was not increased in the SAP group but was increased in the SAP+IL-10 group, indicating that IL-10 activated the STAT3 pathway, which was partly inhibited by S3I-201. Bax expression was up-regulated and Bcl-2 expression was down-regulated in the SAP group compared to the control group. IL-10 reversed the up-regulation of Bax expression and down-regulation of Bcl-2 expression in the SAP group, while S3I-201 partly inhibited the effects of IL-10. Combined with the results of TUNEL staining, we showed that up-regulation of Bax expression and down-regulation of Bcl-2 expression were possible apoptotic mechanisms of BMECs in SAP. Therefore, IL-10 attenuates BMEC apoptosis via the STAT3 pathway by down-regulating the expression of Bax while up-regulating the expression of Bcl-2.

### In vitro experiments

#### Identification of rat BMECs and evaluation of the integrity of constructed in vitro BBB models

**Fig. 4:**
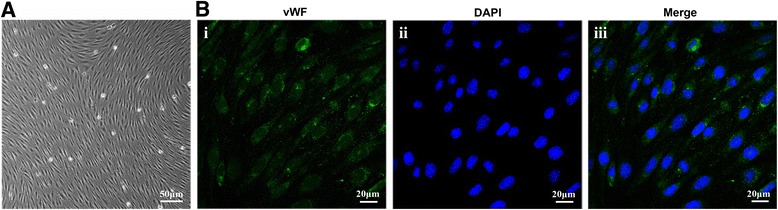
Morphology and identification of rat BMECs. **a** Typical morphology of BMECs; scale bar represents 50 μm. **b** Immunofluorescence of anti-vWF (green) on BMECs; the nuclei were stained with DAPI (blue). Scale bar represents 20 μm

To further ascertain the effects of IL-10 on BMECs under inflammatory conditions and the mechanisms, we established in vitro BBB models with a monolayer of BMECs. Fig. 4A shows a monolayer of non-overlapping, elongated and tightly apposed BMECs. Immunofluorescence staining (Fig. 4B) with a specific endothelial marker, vWF, determined that the purity of puromycin-purified BMECs was approximately 99%, which is in line with the reported data [19]. The TEER value of the in vitro BBB model was 145.0 ± 5.6 Ω × cm^2^, which is similar to that in published data [20, 21], indicating the successful establishment of an in vitro BBB model.

#### IL-10 alleviated the TNF-α-induced breakdown of the in vitro BBB model by maintaining its integrity

**Fig. 5:**
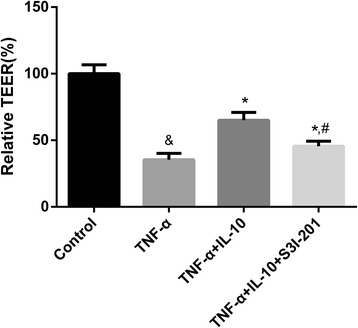
IL-10 alleviated the TNF-α-induced decrease of TEER in BBB models. The control group was set as the calibrator, representing 100%. & *p* < 0.001 (t-test), versus the control group, * *p* < 0.05 (t-test), versus the TNF-α treatment group, # *p* < 0.01 (t-test), versus the TNF-α + IL-10 treatment group

To verify whether IL-10 maintains the integrity of the BBB following TNF-α treatment, we measured the TEER, which is a parameter of BBB integrity. The results of the TEER assay (Fig. 5) showed that TNF-α decreased the TEER value of in vitro BBB models while IL-10 and IL-10 + S3I-201 effectively attenuated the decrease in TEER value, with the IL-10 treatment group exhibiting the highest TEER value. The results indicate that IL-10 maintains the integrity of the in vitro BBB model following TNF-α treatment and that the STAT3 pathway is involved in mediating the protective effects.

#### IL-10 attenuated the TNF-α-induced down-regulation of claudin-5 and impairment of tight junctions in BMECs

**Fig. 6:**
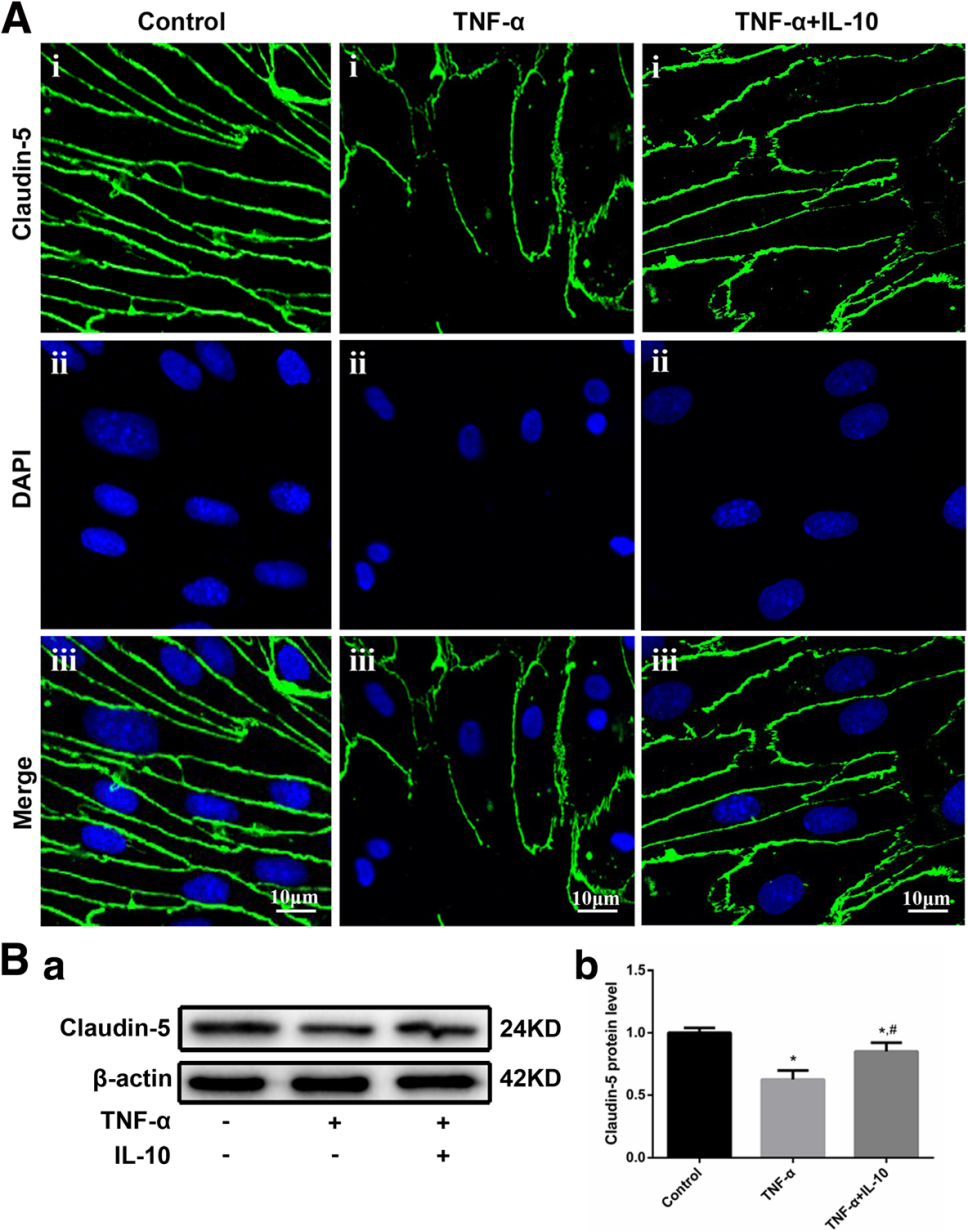
IL-10 attenuated the TNF-α-induced down-regulation of claudin-5 and impairment of tight junctions among BMECs. A. Immunofluorescence staining of claudin-5 (green) on BMECs; the nuclei were stained with DAPI (blue). Scale bar represents 10 μm. Control group showed continuous, linear, smooth peri-cellular tight junctions between BMECs. Tight junctions were extremely disrupted following TNF-α treatment. IL-10 alleviated the impairment of tight junctions induced by TNF-α. B. Representative WB images showing claudin-5 protein expression levels in BMECs (a). WB analysis of claudin-5 expression (*n* = 3, b). * *p* < 0.05 (t-test), versus the control group, # *p* < 0.05 (t-test), versus the TNF-α treatment group

To elucidate the underlying mechanisms of IL-10 in maintaining the integrity of the BBB against TNF-α treatment, we detected changes in claudin-5 expression and the junctional structure by WB and immunofluorescence staining, respectively. Immunofluorescence staining (Fig. 6A) demonstrated that the peri-cellular tight junctions in the control group were continuous, linear and smooth between BMECs. Meanwhile, the tight junctions were severely disrupted following TNF-α treatment, and IL-10 noticeably alleviated this deterioration. The WB results (Fig. 6B) show that TNF-α down-regulated claudin-5 expression in BMECs, which was partly reversed by IL-10. The down-regulation of claudin-5 expression was in accordance with the deterioration of tight junctions. The experimental results thus indicate that IL-10 alleviates the TNF-α-induced down-regulation of claudin-5 and impairment of tight junctions among BMECs and contributes to the maintenance of BBB integrity.

#### IL-10 ameliorated TNF-α-induced BMEC apoptosis by down-regulating Bax and up-regulating Bcl-2 expression via the STAT3 pathway

**Fig. 7:**
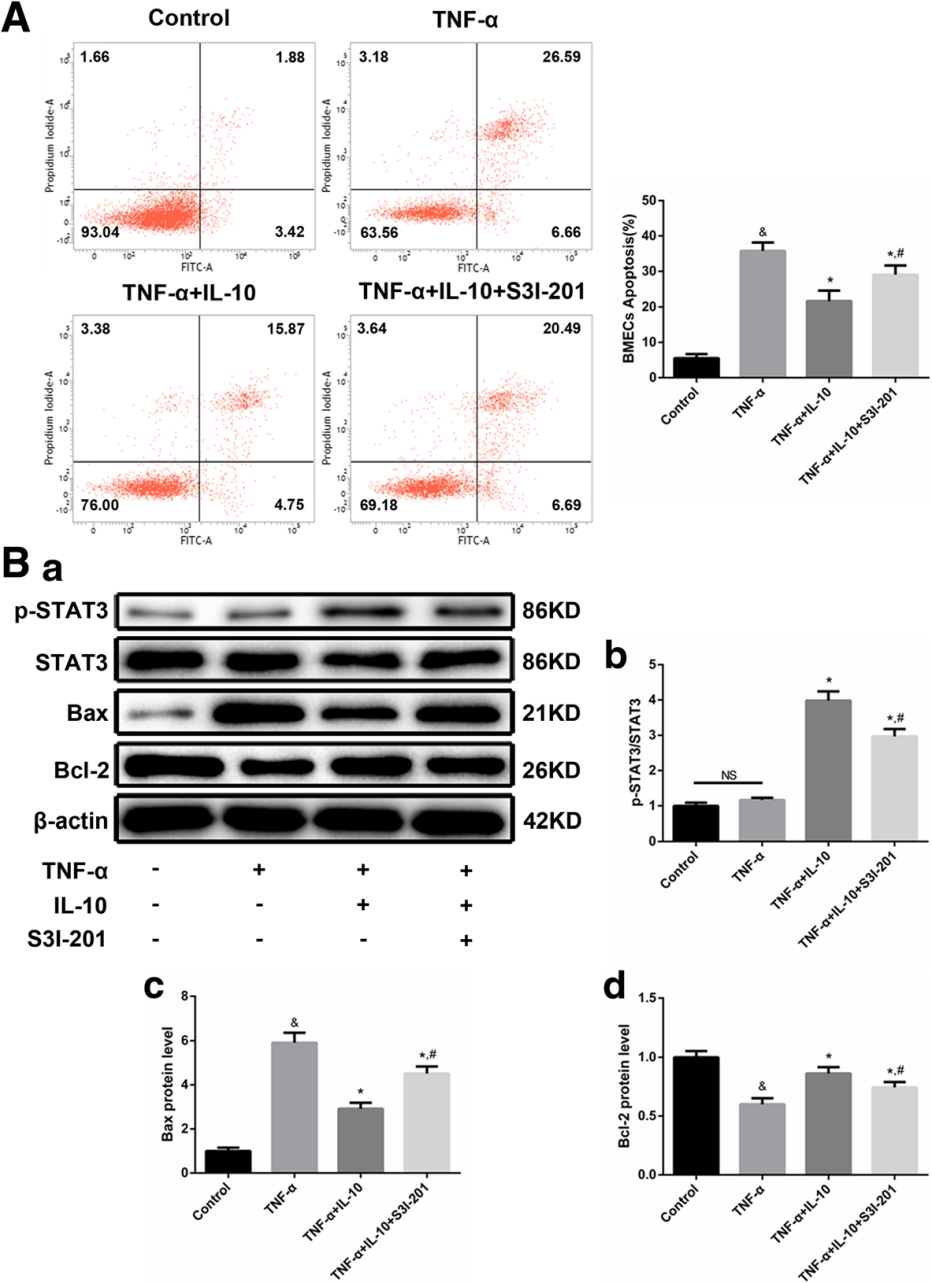
IL-10 ameliorated TNF-α-induced BMEC apoptosis via the STAT3 pathway. A. Assessment of BMEC apoptosis by flow cytometry (*n* = 3). Apoptotic signals in the Q2 and Q4 quadrants were acquired to evaluate BMEC apoptosis. B. Representative WB images of p-STAT3, STAT3, Bax and Bcl-2 protein expression in BMECs (a). WB analysis of p-STAT3/STAT3 expression (*n* = 3, b). WB analysis of Bax expression (*n* = 3, c). WB analysis of Bcl-2 expression (*n* = 3, d). & *p* < 0.001 (t-test), versus the control group, * *p* < 0.05 (t-test), versus the TNF-α treatment group, # *p* < 0.05 (t-test), versus the TNF-α + IL-10 treatment group. NS: no statistical difference

To verify the anti-apoptotic effect of IL-10 on BMECs and the involvement of the STAT3 pathway in the in vivo experiments, we detected TNF-α-induced BMEC apoptosis by flow cytometry and the expression of downstream proteins in the STAT3 pathway by WB. Flow cytometry data (Fig. 7A) showed that TNF-α significantly increased BMEC apoptosis, whereas IL-10 noticeably reduced this increase in BMEC apoptosis. In addition, S3I-201 partially reversed the IL-10-mediated anti-apoptotic effect.

WB results (Fig. 7B) showed low expression levels of p-STAT3 in both the control and TNF-α treatment groups. A significantly higher level of p-STAT3 expression was observed in the IL-10 treatment group than in the control group, indicating STAT3 pathway activation. Meanwhile, S3I-201 markedly decreased p-STAT3 expression by inhibiting STAT3 phosphorylation, and TNF-α significantly increased Bax expression and decreased Bcl-2 expression, thus resulting in BMEC apoptosis. IL-10 noticeably reversed this expression tendency, eliciting anti-apoptotic effects, which were attenuated by S3I-201. In summary, in line with the in vivo experiments, IL-10 alleviated TNF-α-induced BMEC apoptosis via the STAT3 pathway by down-regulating Bax expression and up-regulating Bcl-2 expression.

## Discussion

SAP is a classical acute critical inflammatory disease with cascade reactions and often results in organ dysfunction. SAP accompanied with brain dysfunction is defined as pancreatic encephalopathy, which usually has psychosomatic manifestations and a high mortality rate. There are many hypotheses surrounding the underlying pathogenesis of pancreatic encephalopathy; however, it is widely accepted that impairment of the BBB greatly contributes to brain dysfunction [22–24]. Previous studies have revealed that inflammation induces BMEC apoptosis and the deterioration of tight junctions, thereby increasing BBB permeability [25]. Consequently, protecting BMECs and peri-cellular tight junctions from inflammation is simultaneously beneficial for the maintenance of BBB stability and prevention of pancreatic encephalopathy.

IL-10 has been extensively studied due to its great immunoregulatory and anti-inflammatory abilities. Published reports show that IL-10 attenuates the inflammatory response and decreases TNF-α secretion in acute pancreatitis [26, 27]. It was verified in our study that IL-10 alleviates the severity of inflammation in SAP by decreasing the pancreatic pathological score as well as serum amylase and TNF-α secretion. Furthermore, IL-10 protects the BBB from increased permeability in SAP rats, as confirmed by the Evan’s blue assay and TEER measurements. The IL-10-induced decrease in TNF-α secretion might be helpful in stabilizing the BBB, as TNF-α has been reported to increase BBB permeability in acute pancreatitis [28]. To further explore the protective effects of IL-10 on the BBB under inflammatory conditions and the underlying mechanisms, we conducted a series of experiments to analyze the changes in BMECs and peri-cellular tight junctions. Additionally, in vitro BBB models consisting of a monolayer of BMECs were constructed to further confirm the results of the in vivo experiments.

Claudin-5 is a key component of tight junction proteins and is mainly expressed on BMECs in the brain. Claudin-5 controls BBB permeability and integrity, and its expression level is more than 100-fold that of other claudins [29]. Previous studies have demonstrated that claudin-5 expression is down-regulated [30, 31] and that junctional structure is deteriorated [32] under inflammatory conditions, which were also observed in our study. Meanwhile, we found that IL-10 attenuated the TNF-induced down-regulation of claudin-5 and impairment of tight junctions. The mechanisms remain unclear, but possible explanations include the following: IL-10 decreased TNF-α secretion; IL-10 attenuated TNF-α-induced BMEC apoptosis, thus implying enhanced transcription and translation of claudin-5; and IL-10 down-regulated TNF-α receptor expression or inhibited the binding of TNF-α with its receptor.

BMECs are the functional parts of the BBB, and impaired BMECs lead to BBB dysfunction. Inflammation, hypoxia, hemorrhage and pharmaceutical agents have been shown to induce BMEC apoptosis, especially inflammation [33–36]. In animal experiments, we observed plentiful BMEC apoptosis and some glial cell apoptosis in the brain of SAP rats at 12 h. However, fewer BMECs and no glial cells were apoptotic in SAP rats treated with IL-10. This result may be attributed to the anti-apoptotic effects of IL-10 on BMECs and the protective effects of IL-10 on tight junctions, which help maintain BBB integrity and protect glial cells from inflammatory cytokines via an indirect anti-apoptotic effect. To confirm the anti-apoptotic effects of IL-10 on BMECs and the potential mechanisms in the in vivo experiments, we administered TNF-α to BBB models to induce inflammation. We found that IL-10 remarkedly attenuated the TNF-α-induced BMEC apoptosis, junctional discontinuity and down-regulation of claudin-5 expression. The experimental results further confirm that IL-10 maintains BBB integrity under inflammatory situations.

The anti-apoptotic effects of IL-10 on BMECs under inflammatory conditions were confirmed by both in vivo and in vitro experiments; however, the underlying mechanisms remain unclear. Previous studies have shown that IL-10 improves endothelial progenitor cell survival and function under hypoxic conditions via the STAT3/VEGF signaling pathway [37]. In addition, IL-10 overexpression improves endothelial progenitor cell function following TNF-α stimulation via the STAT3 pathway [38]. To date, the IL-10/STAT3 signaling pathway has been reported to play an important part in tumor progression, chemotherapy resistance [39] and the anti-inflammatory response. The STAT3 pathway is activated by the binding of IL-10 to the IL-10 receptor. Following activation, STAT3 is phosphorylated, and p-STAT3 is transferred to the nucleus to modulate target protein expression, including Bax and Bcl-2, which are important for cell apoptosis. Previous studies have demonstrated that Bax promotes TNF-α-induced endothelial cell apoptosis via mitochondrial apoptotic signaling pathways, whereas Bcl-2 exhibits anti-apoptotic ability by stabilizing mitochondrial membrane potential [40]. Adding to the existing findings, we hypothesized that IL-10 might elicit anti-apoptotic effects on BMECs by activating the STAT3 pathway and modulating Bax and Bcl-2 expression. Consistent with previously published reports, we observed that both SAP and TNF-α treatment significantly increased Bax expression and decreased Bcl-2 expression, resulting in BMEC apoptosis, which was confirmed by TUNEL staining and flow cytometry. We also found that exogenous IL-10 indeed activated the STAT3 pathway and attenuated the up-regulation of Bax and down-regulation of Bcl-2 expression, resulting in anti-apoptotic effects on BMECs under inflammatory conditions. Administration of the STAT3 inhibitor S3I-201 partly blocked the IL-10-mediated anti-apoptotic effects, further confirming the involvement of the STAT3 pathway.

Clinical trials have reported that IL-10 is effective in treating inflammatory diseases [41], including Crohn’s disease and rheumatoid arthritis. Further exploration of the underlying mechanisms of the IL-10 signaling pathway or the modulation of IL-10 expression might lead to the development of new therapeutic modalities for inflammatory diseases. In the present study, we found that IL-10 attenuated impairment of the BBB in SAP. However, many mechanisms remain unclear, including the underlying mechanisms by which IL-10 attenuates the down-regulation of claudin-5 expression and impairment of junctional structure under inflammatory situations. Additional research will be required to elucidate these mechanisms in the future.

## Conclusions

Our study demonstrated that IL-10 attenuated the severity of inflammation and increased BBB permeability in SAP models as well as stabilized the integrity of in vitro BBB models following TNF-α treatment. The underlying mechanisms of IL-10 include the alleviation of down-regulated claudin-5 expression and impaired junctional structure and the attenuation of BMEC apoptosis via the STAT3 pathway by decreasing Bax expression while increasing Bcl-2 expression. Our results confirm the protective effects of IL-10 on BMECs under inflammatory conditions and the possible mechanisms, which provides a novel modality for the prevention and treatment of pancreatic encephalopathy that may be tested in prospective clinical trials.

## Abbreviations

BBB: Blood-brain barrier
BMECs: Brain microvascular endothelial cells
IL-10: Interleukin-10
SAP: Severe acute pancreatitis
STAT3: Signal transducers and activators of transcription 3
TNF-α: Tumor necrosis factor-α
